# Intracholecystic papillary-tubular neoplasm in a patient with choledochal cyst: a link between choledochal cyst and gallbladder cancer?

**DOI:** 10.1186/s12957-016-0962-x

**Published:** 2016-08-02

**Authors:** Adamantios Michalinos, Parakevi Alexandrou, Alexandros Papalambros, Dimitrios Oikonomou, Stratigoula Sakellariou, Evangelia Baliou, Andreas Alexandrou, Dimitrios Schizas, Evangelos Felekouras

**Affiliations:** 1 First Department of Surgery, National and Kapodistrian University of Athens, Ag. Thoma 17 Str., Goudi, Athens, Greece; 2 First Department of Pathology, National and Kapodistrian University of Athens, Athens, Greece

**Keywords:** Intracholecystic papillary neoplasm, Choledochal cyst, Gallbladder cancer

## Abstract

**Background:**

Intracholecystic papillary-tubular neoplasms are rare precursor lesions of gallbladder cancer. They were proposed as a separate pathologic entity in 2012 by Adsay et al. for the unification of a variety of mass-forming precursor lesions including papillary adenomas, tubulopapillary adenomas, intestinal adenomas, and others. They are considered homologous to intrapapillary mucinous neoplasms of the pancreas and intrabiliary papillary neoplasms of the common bile duct. In contrast with the commoner flat-type precursor gallbladder cancer lesions, they follow a more indolent clinical course and probably different genetic pathways to carcinogenesis. They are largely uninvestigated with only a handful of studies providing biological and clinical information. Choledochal cysts are dilation of the common bile duct. Diagnosis is usually established during childhood, and only a minority of patients are diagnosed at adulthood. They are of major clinical importance as they are known predisposing factors for biliary carcinogenesis.

**Case presentation:**

The current report describes a patient with a simultaneous diagnosis of choledochal cyst and intracholecystic papillary-tubular neoplasm. The patient underwent excision of the extrahepatic biliary tree for a Todani I choledochal cyst, and histological examination of the specimen revealed an intracholecystic papillary-tubular neoplasm of the gallbladder. Authors describe diagnostic and clinical course of the patient alongside clinical and biological characteristics of these rare lesions.

**Conclusions:**

To the best of our knowledge, this is the first report of a patient with a simultaneous diagnosis of choledochal cyst and intracholecystic papillary-tubular neoplasm. Those rare lesions shed light on different forms of gallbladder cancer carcinogenesis and its relationship with choledochal cysts and cholestasis.

## Background

Precursor lesions of gallbladder cancer separate into two categories: those exhibiting flat dysplasia and the mass-forming lesions. The former are commoner and proceed to carcinogenesis through a metaplasia-dysplasia cancer pathway [1, 2]. The latter have been described under a variety of names including papillary adenomas, tubulopapillary adenomas, intestinal adenomas, and others [3]. The World Health Organization 2010 classification distinguishes two categories, the “adenomas” and the “intracystic papillary neoplasms” [4] but without providing specific diagnostic criteria and thus allowing significant overlapping. In 2012, Adsay et al. [5] proposed unification of those lesions under the category of “intracholecystic papillary-tubular neoplasm” (ICPN) identifying common morphological, immunohistochemical and clinical characteristics. Furthermore, they proposed homology with the more extensively described intraductal papillary mucinous neoplasms (IPMNs) of the pancreas and the recently described intraductal biliary mucinous neoplasms (IPBNs) of the bile ducts. They state that those neoplasms follow an adenoma-carcinoma sequence, different from the metaplasia-dysplasia-carcinoma sequence, establishing an alternative model for gallbladder carcinogenesis.

ICPNs are rare neoplasms of the gallbladder, homologous to pancreatic IPMNs, and the recently described IPBNs. Adsay et al. [5] define them as tumors composed of preinvasive neoplastic cells that form clinically detectable masses >1.0 cm [5]. This definition unifies a wide spectrum of previously described neoplastic and preneoplastic lesions of the gallbladder including papillary adenomas, tubulopapillary adenomas, intestinal adenomas, biliary adenomas, transitional adenomas, papillary neoplasms, papillary carcinomas, and intracystic papillary neoplasm. Their definition as a single category derives from their common clinicopathological, immunophenotypic and molecular characteristics, their better prognosis, and homology to other papillary neoplasms of the pancreatobiliary track, including pancreatic IPMNs and biliary IPBNs [3]. The criterion of 1.0 cm is arbitrary yet relevant since tumors <1 cm are usually undetectable preoperatively and almost universally benign [6, 7].

Since their first description in 2012 by Asday et al. [5], only two series by Isozaki et al. [8] and Bennet et al. [9] encompassing 23 and seven cases, respectively, and some case reports have been published [2, 10–13] (Table 1). Undoubtedly, ICPNs have been described in older series [14], yet adoption of specific diagnostic criteria only in 2012 makes their retrospective study very difficult. In fact, the 2010 World Health Classification recognizes two generic categories, adenomas and intracystic papillary neoplasms, with a variety of subcategories without providing specific diagnostic criteria and allowing significant overlapping [4].

**Table 1:** Series in the literature describing ICPNs

Author	No	Patients	Size	Invasive	Subtype	T stage^a^	Survival	
							Noninvasive	Invasive
Adsay et al. [5] 2012	123	F/M: 2/1 Mean age: 61 years	2.6 cm	44.7 %	Biliary: 50 % Gastric: 36 % Intestinal: 10.8 % Oncocytic: 8.6 %	T1: 32 % T2: 47 % T3: 21 %	1 year: 90 % 3 years: 90 % 5 years: 58 %	1 year: 69 % 3 years: 60 % 5 years: 60 %
Isozaki et al. [8] 2014	23	F/M: 9/14 Mean age: 69 years	2.8 cm	39.1 %	Biliary: 56.5 % Gastric: 34.7 % Intestinal: 8.7 %	T1: 36 % T2: 50 % T3: 14 %	3 years: 91 % 5 years: 67 %	
Bennet et al. [9] 2015	7	F/M: 6/1 Mean age: 68 years	6.2 cm	42.9 %	Biliary: 71.4 % Gastric: 14.2 % Intestinal: 14.2 %	T1: 25 % T2: 25 % T3: 50 %	3 years: 71 % 5 years: 58 %	

^a^Applies only to invasive disease

ICPNs intracholecystic papillary neoplasms

ICPNs are considered analogous to IPBNs and to a lesser extent to IPMNs. This is supported by their exophytic nature, expression of the same cellular lineages, similar clinical behavior, and better survival. Furthermore, ICPNs seem to progress to cancer through an adenoma-carcinoma sequence rather than the much commoner metaplasia-dysplasia-carcinoma sequence [5, 8]. Until today, no molecular studies have revealed specific genetic pathways. Nevertheless, there are differences between ICPNs and IPMNs like expression of a dominant pancreaticobiliary phenotype and normal GNAS gene expression [11].

Macroscopically, they are polypoid lesion presenting large villous/papillary growths with smooth surface projections. They can be sessile or pedunculated [3]. According to Adsay et al. [5], ICPNs can be of papillary (43 %), tubular (23 %), or mixed (31 %) configuration and of biliary (50 %), gastric (36 %), intestinal (11 %), or oncocytic (9 %) cellular lineage. Biliary type ICPNs commonly express MUC1, a marker of biliary differentiation; gastric type ICPNs express MUC5AC; intestinal type ICPNs express CK20; and oncocytic type ICPNs express HepPar. Mixed forms are common. Biliary phenotype and MUC1 expression are bad prognostic factors, correlated to progression to carcinoma and worse prognosis [5, 9].

A more benign clinical behavior than flat type precursor lesions characterizes ICPNs. ICPNs are incidentally found at only 0.4 % of cholecystectomies. Approximately 6.4 % of gallbladder cancers carry an ICPN component, indicating that ICPNs can progress to gallbladder cancer. Stage-matched comparison between typical gallbladder cancer and invasive ICPNs proved that ICPNs metastasize less often to lymph nodes and have better disease-free and overall survival [5, 8, 13]. ICPN component at invasive cancer usually is of papillary configuration and has a more extensive component of high-grade dysplasia, indicating a stepwise progression to carcinoma [5]. The difference in prognosis can be attributed to their exophytic nature causing earlier symptoms and earlier diagnosis or an inherent more indolent biology [3, 8, 9, 13]. Notably, similar conclusions have been reached for IPBNs [15].

Choledochal cysts are rare congenital dilatations of the biliary track. Although benign, their presence is associated with serious complications including biliary carcinoma, pancreatitis, and choledocholithiasis [16]. Their incidence in the Western population is estimated at 1/100.00 and Japanese population at 1/1000 [17, 18]. They are classified into five types according to the Todani classification: [19] type I: fusiform cystic dilation of the extrahepatic biliary tree (80 %), type II: bile duct diverticulum, (3 %), type III choledochocele (4 %), type IV: Multiple extrahepactic bile duct dilatations (13 %), and type V: Caroli’s disease (1 %) [20].

Choledochal cysts are predominantly present in children. They are four times commoner in females. In developed countries, only 20–30 % of the patients are diagnosed at adulthood [16]. Patients carry a significant risk for biliary track cancer, with incidence increasing with age <1 % in patients younger than 10 years, 15 % in patients older than 20 years, and up to 35 % in patients >60 years [18, 21]. Seventy percent of the tumors are located in the bile ducts and 30 % in the gallbladder [18]. Excision of the extrahepatic biliary tree with the creation of a Roux-en-Y hepaticojejunostomy is the best treatment option for types I and IV. Diverticulotomy with primary common bile ducts closure is usually sufficient for type II, while type V requires orthotopic liver transplantation as treatment. All types of choledochal cyst, especially I and IV are known predisposing factors for cholangiocarcinoma and to a lesser extent for gallbladder carcinoma [16, 22].

Etiology of choledochal cysts is unknown. Their pathophysiology is closely related to the existence of pancreatic maljunction, i.e., abnormal junction of the common biliary duct and pancreatic duct outside the duodenal wall. It is a rare disorder, occurring in less than 2 % of the population while >80 % of all pediatric choledochal cysts are associated with pancreatobiliary maljunction [16, 23]. Sphincter of Oddi cannot prevent regurgitation and a premature mixture of bile and pancreatic juice into the common bile duct or the pancreatic duct, leading to improper activation of pancreatic enzymes, deconjugation of bile salts and subsequent cholestasis and chronic inflammation. Amylase levels at common bile duct are elevated in patients with pancreatobiliary maljunction [16]. Pancreatobiliary maljunction is a known predisposing factor for both cholangiocarcinoma and gallbladder carcinoma and, to a lesser extent, pancreatic carcinoma [16, 18, 24, 25].

## Case presentation

**Fig. 1:**
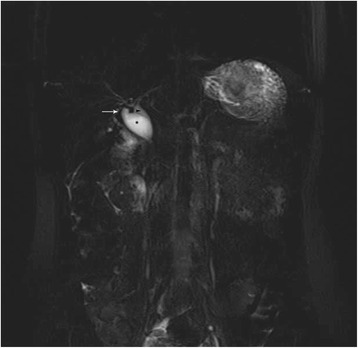
Preoperative magnetic resonance cholangiopancreatography showing a type I Todani classification choledochal cyst. *Arrow* cystic duct, *arrowhead* common bile duct, *choledochal cyst

A young Caucasian female patient aged 28 years came to our hospital complaining of chronic right upper quadrant pain. Physical examination was unremarkable as was her medical history. She was investigated with upper abdominal ultrasound that was negative for chololithiasis but revealed dilated gallbladder and common bile duct with a diameter of 2.1 cm. Her laboratory values were within normal limits with total bilirubin of 0.28 mg/dl, alkaline phosphatase (ALP) at 42 U/l, and γ- GT 16 U/l. Serum tumor markers were within normal limits. The patient was further studied with computerized tomography, magnetic resonance imaging, and magnetic resonance cholangiopancreatography (Fig. 1) that proved dilation of common bile duct between the confluence of hepatic ducts down to the upper pancreatic border. The intrapancreatic portion was normal. The investigation was negative for choledocholithiasis and otherwise unremarkable. Pancreaticobiliary junction was normal.

**Fig. 2:**
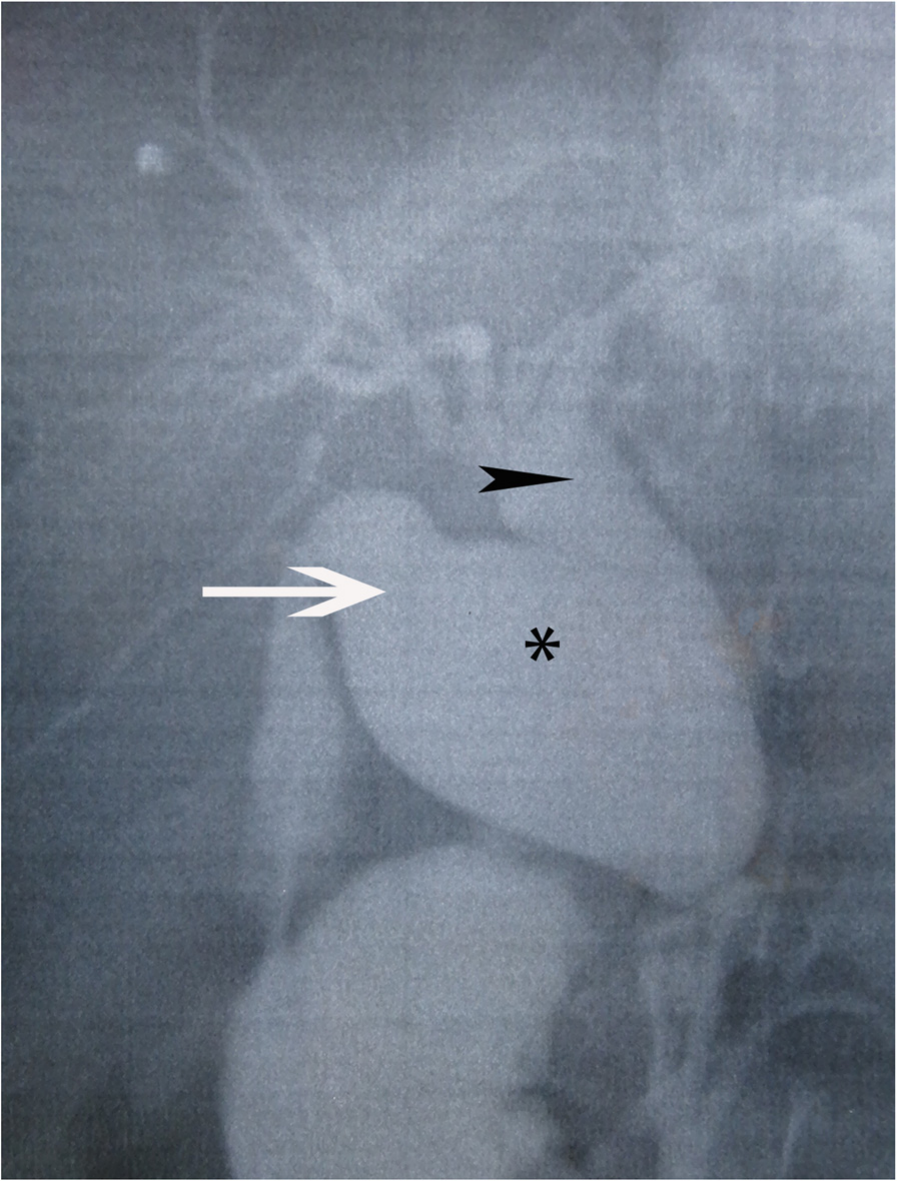
Intraoperative cholangiography of the patient showing a type I Todani classification choledochal cyst. *Arrow* cystic duct, *arrowhead* common bile duct, *choledochal cyst

With a diagnosis of a choledochal cyst, Type Ia according to Todani classification, the patient underwent excision of the extrahepatic biliary tree with Roux-en-Y reconstruction. An intraoperative cholangiography was performed for diagnosis confirmation (Fig. 2).

**Fig. 3:**
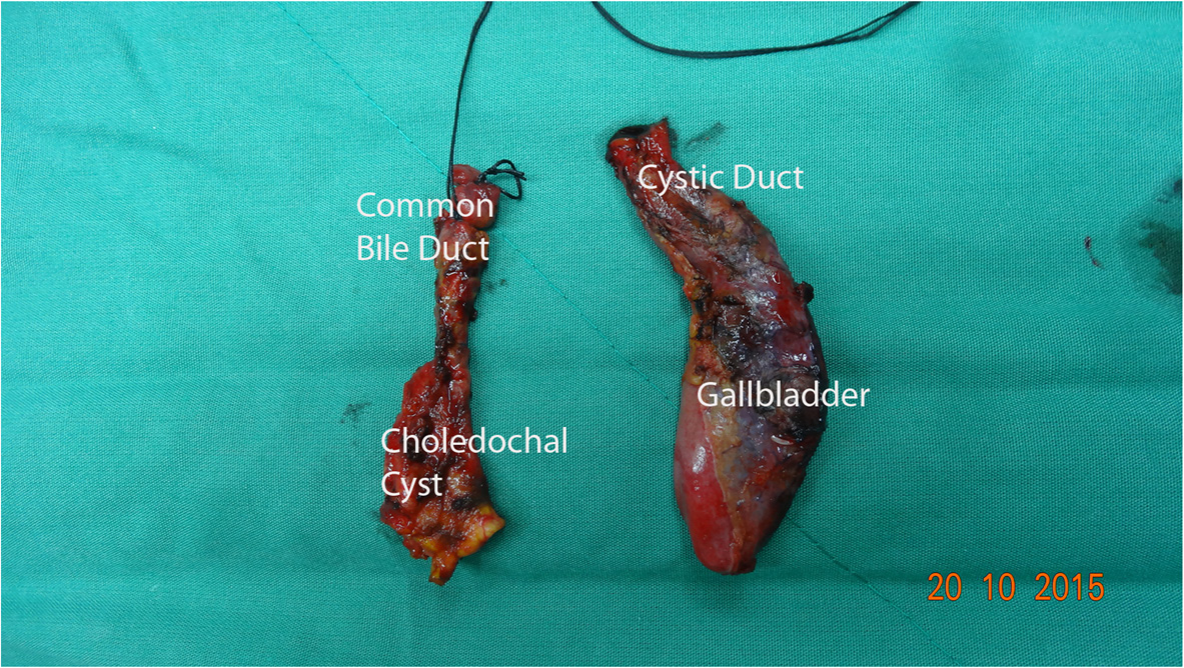
Macroscopic image of the specimen showing a type I choledochal cyst and a dilated cystic duct

**Fig. 4:**
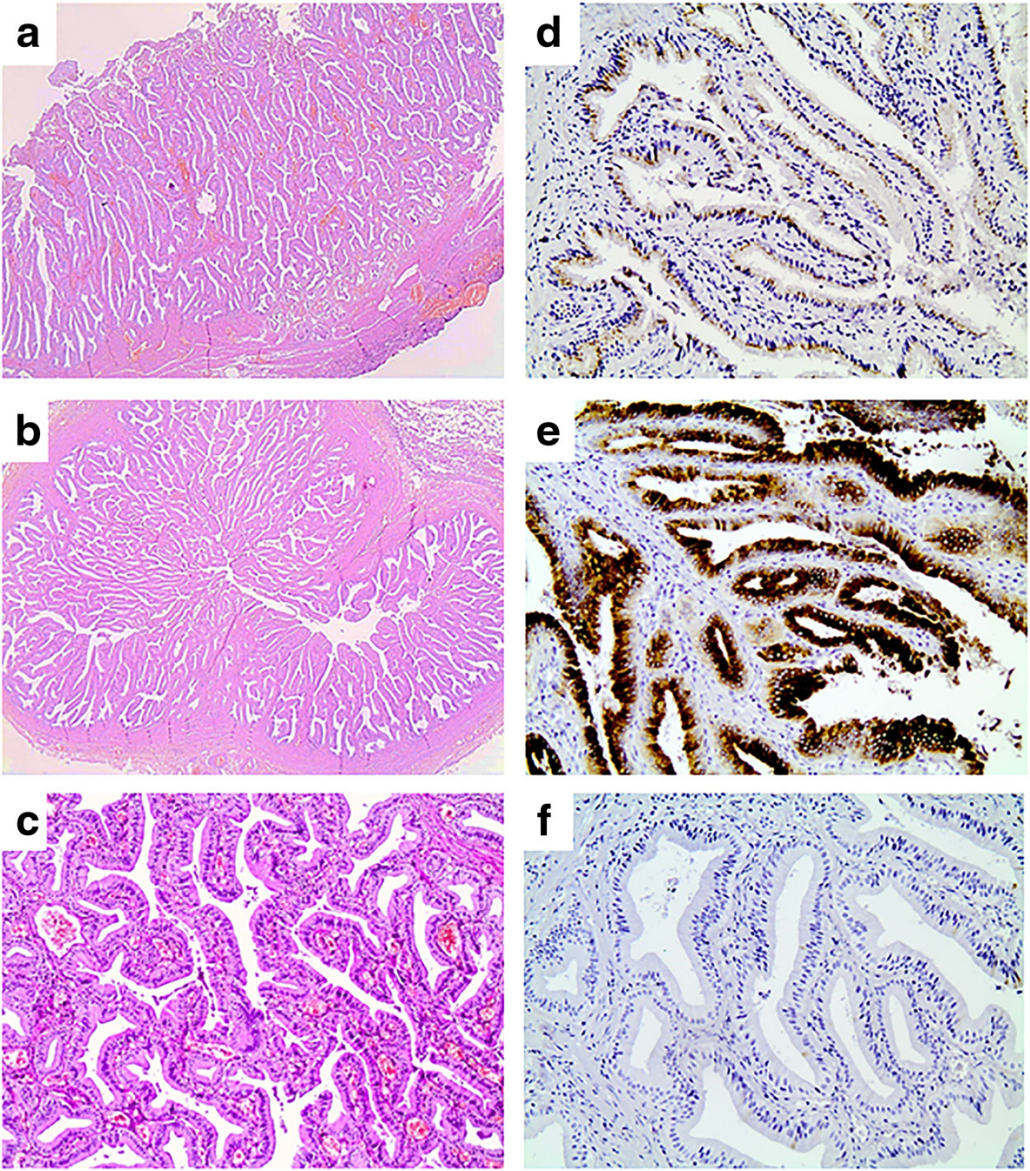
Histological and immunohistochemical features of the neoplasm. **a** Prominent intraluminal papillary proliferation of the gallbladder (×20). **b** A papillary neoplasm is filling the lumen of the cystic duct (×20). **c** Pancreatobiliary-type epithelium with mild nuclear pseudostratification (×100). **d** MUC1 expression (×200). **e** MUC5AC expression (×200). **f** MUC2 negativity (×200)

The patient had an uneventful recovery and left the hospital at the seventh postoperative day. Specimen was sent for histological examination (Fig. 3). On histology, the gallbladder, the cystic duct, and partly the common bile duct showed a prominent intraluminal papillary proliferation of neoplastic epithelial cells with delicate fibrovascular stalks. Papillary epithelium consisted of columnar cells with eosinophilic cytoplasm and round nuclei with minimal irregularities and mild pseudostratification, resembling slightly dysplastic biliary epithelium. Immunohistochemical staining revealed MUC1 and MUC5AC expression while tumor cells were negative for MUC2. Findings were consistent with intracystic papillary neoplasm of the gallbladder and intraductal papillary neoplasm of the cystic and common bile duct with low-grade dysplasia, pancreatobiliary subtype (Fig. 4).

## Conclusions

In this report, authors describe a rare case of ICPN incidentally discovered after excision of the extrahepatic biliary tree for a type I choledochal cyst in a young female patient. We also perform a brief literature review on ICPNs and mechanisms of gallbladder carcinogenesis. To the best of our knowledge, this is the first time an ICPN was reported in a patient with a choledochal cyst.

Resende et al. [10] reported a simultaneous diagnosis of an ICPN and an IPBN in the same patient. This patient also presented pancreaticobiliary maljunction, a common etiologic factor for both gallbladder cancer and cholangiocarcinoma. Similarly, Kasuya et al. reported a patient with pancreaticobiliary maljunction and simultaneous diagnosis of gallbladder cancer and cholangiocarcinoma [26].

Those rare precancerous lessions give insight to carcinogenesis of biliary track cancer. Their rarity does not allow concrete conclusion formulation; still, few accumulated evidence indicates a relationship between extrahepatic obstruction and biliary stasis and carcinogenesis in the biliary track. Biliary stasis causes continuous inflammation and epithelium damage, leading the biliary epithelium to regenerative cycles. Inevitable acceleration of the cell cycle makes it susceptible to metaplasia and consequent dysplasia. It seems that this pathophysiological mechanism also causes ICPNs, yet why some patients will follow the more malignant metaplasia-dysplasia-carcinoma pathway and others the more indolent adenoma-carcinoma pathway remains unknown [1, 27].

## Abbreviations

ALP, alkaline phosphatase; ICPN, intracholecystic papillary-tubular neoplasm; IPBN, intraductal papillary biliary neoplasm; IPMN, intraductal papillary mucinous neoplasm; γ-GT, gamma-glutamyl transferase

## References

[1] Roa I, de Aretxabala X, Araya JC, Roa J (2006). Preneoplastic lesions in gallbladder cancer. J Surg Oncol.

[2] Dekate J, Serra S, Chetty R (2015). Intracholecystic papillary-tubular neoplasm. Diagn Histopathol.

[3] Xue Y, Quigley B, Akkas G, Adsay V (2015). Neoplastic precursors (dysplasia, intraepithelial neoplasia) of the gallbladder and biliary tract: terminology, classification, pathologic diagnosis, and clinical significance. Diagnost Histopath.

[4] Albores-Saavedra J, Adsay N, Crawford J (2010). Carcinoma of the gallbladder and the extrahepatic bile ducts. World Health Organization Classification of Tumors Tumors of Digestive System.

[5] Adsay V, Jang K-T, Roa JC, Dursun N, Ohike N, Bagci P (2012). Intracholecystic papillary-tubular neoplasms (ICPN) of the gallbladder (neoplastic polyps, adenomas, and papillary neoplasms that are ≥1.0 cm): clinicopathologic and immunohistochemical analysis of 123 cases. J Surg Pathol.

[6] Lee CH, Giurescu ME, Philpotts LE, Horvath LJ, Tocino I (1997). Clinical importance of unilaterally enlarging lymph nodes on otherwise normal mammograms. Radiology.

[7] Yang HL, Sun YG, Wang Z (1992). Polypoid lesions of the gallbladder: diagnosis and indications for surgery. Br J Surg.

[8] Isozaki M, Ohike N, Mitsuya T, Takimoto M (2014). Clinicopathological study of intracystic papillary-tubular neoplasms (ICPN) of the gallbladder. Showa Univ J Med Sci.

[9] Bennett S, Marginean EC, Paquin-Gobeil M, Wasserman J, Weaver J, Mimeault R (2015). Clinical and pathological features of intraductal papillary neoplasm of the biliary tract and gallbladder. HPB (Oxford).

[10] Resende V, Roda R, Pedrosa M (2012). Gallbladder papillary neoplasia associated with intrahepatic carcinoma and pancreaticobiliary malformation. Gastroenterol Res.

[11] Hashimoto S, Horaguchi J, Fujita N, Noda Y, Kobayashi G, Ito K (2014). Intracholecystic papillary-tubular neoplasm of the gallbladder presenting with jaundice. Intern Med.

[12] Yamamoto K, Yamamoto F, Maeda A, Igimi H, Yamamoto M, Yamaguchi R (2014). Tubulopapillary adenoma of the gallbladder accompanied by bile duct tumor thrombus. World J Gastroenterol.

[13] You JS, Chung SP, Park JY, Park S, Chung TN, Park I (2012). The utility of the HeartSaver Sticker for maintaining correct hand position during chest compressions. J Emerg Med.

[14] Albores-Saavedra J, Tuck M, McLaren BK, Carrick KS, Henson DE (2005). Papillary carcinomas of the gallbladder: analysis of noninvasive and invasive types. Arch Pathol Lab Med.

[15] Serra S (2014). Precursor neoplastic lesions of the biliary tract. J Clin Pathol.

[16] Soares KC, Arnaoutakis DJ, Kamel I, Rastegar N, Anders R, Maithel S (2014). Choledochal cysts: presentation, clinical differentiation, and management. J Am Coll Surg.

[17] Singham J, Yoshida EM, Scudamore CH (2009). Choledochal cysts. Can J Surg.

[18] Sastry AV, Abbadessa B, Wayne MG, Steele JG, Cooperman AM (2015). What is the incidence of biliary carcinoma in choledochal cysts, when do they develop, and how should it affect management?. World J Surg.

[19] Todani T, Watanabe Y, Narusue M, Tabuchi K, Okajima K (1977). Congenital bile duct cysts: classification, operative procedures, and review of thirty-seven cases including cancer arising from choledochal cyst. Am J Surg.

[20] Nagorney DM, McIlrath DC, Adson MA (1984). Choledochal cysts in adults: clinical management. Surgery.

[21] Andsay N, Klimstra D (2009). Bening and malignant tumors of the gallbladder and the extrahepatic biliary track. Surgical Pathology of the GI Tract, Liver, Biliary tract, and Pancreas.

[22] Rustagi T, Dasanu CA (2012). Risk factors for gallbladder cancer and cholangiocarcinoma: similarities, differences and updates. J Gastrointest Cancer.

[23] Korea J (2012). Cancer of the bile ducts. Blumgart’s surgery of the liver, biliary tract and pancreas.

[24] Funabiki T, Matsubara T, Miyakawa S, Ishihara S (2009). Pancreaticobiliary maljunction and carcinogenesis to biliary and pancreatic malignancy. Langenbecks Arch Surg.

[25] Katabi N, Pillarisetty VG, DeMatteo R, Klimstra DS (2014). Choledochal cysts: a clinicopathologic study of 36 cases with emphasis on the morphologic and the immunohistochemical features of premalignant and malignant alterations. Hum Pathol.

[26] Kasuya K, Nagakawa Y, Matsudo T, Ozawa T, Tsuchida A, Aoki T (2009). p53 gene mutation and p53 protein overexpression in a patient with simultaneous double cancer of the gallbladder and bile duct associated with pancreaticobiliary maljunction. J Hepatobiliary Pancreat Surg.

[27] Toledo C, Matus CE, Barraza X, Arroyo P, Ehrenfeld P, Figueroa CD (2012). Expression of HER2 and bradykinin B_1_ receptors in precursor lesions of gallbladder carcinoma. World J Gastroenterol.

